# Predicting the metabolic cost of exoskeleton-assisted squatting using foot pressure features and machine learning

**DOI:** 10.3389/frobt.2023.1166248

**Published:** 2023-04-19

**Authors:** Sruthi Ramadurai, Heejin Jeong, Myunghee Kim

**Affiliations:** ^1^ Rehabilitation Robotics Laboratory, Department of Mechanical and Industrial Engineering, University of Illinois at Chicago, Chicago, IL, United States; ^2^ The Polytechnic School, Ira A. Fulton Schools of Engineering, Arizona State University, Mesa, AZ, United States

**Keywords:** lower limb exoskeleton, personalized assistance, foot pressure, center of pressure, metabolic cost, machine learning

## Abstract

**Introduction:** Recent studies found that wearable exoskeletons can reduce physical effort and fatigue during squatting. In particular, subject-specific assistance helped to significantly reduce physical effort, shown by reduced metabolic cost, using human-in-the-loop optimization of the exoskeleton parameters. However, measuring metabolic cost using respiratory data has limitations, such as long estimation times, presence of noise, and user discomfort. A recent study suggests that foot contact forces can address those challenges and be used as an alternative metric to the metabolic cost to personalize wearable robot assistance during walking.

**Methods:** In this study, we propose that foot center of pressure (CoP) features can be used to estimate the metabolic cost of squatting using a machine learning method. Five subjects’ foot pressure and metabolic cost data were collected as they performed squats with an ankle exoskeleton at different assistance conditions in our prior study. In this study, we extracted statistical features from the CoP squat trajectories and fed them as input to a random forest model, with the metabolic cost as the output.

**Results:** The model predicted the metabolic cost with a mean error of 0.55 W/kg on unseen test data, with a high correlation (r = 0.89, *p* < 0.01) between the true and predicted cost. The features of the CoP trajectory in the medial-lateral direction of the foot (xCoP), which relate to ankle eversion-inversion, were found to be important and highly correlated with metabolic cost.

**Conclusion:** Our findings indicate that increased ankle eversion (outward roll of the ankle), which reflects a suboptimal squatting strategy, results in higher metabolic cost. Higher ankle eversion has been linked with the etiology of chronic lower limb injuries. Hence, a CoP-based cost function in human-in-the-loop optimization could offer several advantages, such as reduced estimation time, injury risk mitigation, and better user comfort.

## 1 Introduction

### 1.1 Background

Workers frequently perform movements such as squatting and lifting during manual operations in industrial sites (Jeong et al., 2020). Often, these squat lifting movements are repetitive and lead to physical fatigue, increasing the risk of injuries such as musculoskeletal disorders, low back pain and arthritis (Andersen et al., 2007; Balasubramanian et al., 2009; McDonough and Jette, 2010; Werner et al., 2010; Bergmann et al., 2017; Breloff et al., 2019). These are major issues that affect the workers’ occupational health and reduce their quality of life (Briggs et al., 2016). Recent studies have recommended the use of wearable exoskeletons to reduce physical effort and fatigue during repetitive squatting (Gams et al., 2013; Petrič et al., 2013; Mohri et al., 2017; Ranaweera et al., 2018; Sado et al., 2019; Jeong et al., 2020; Jeong et al., 2023; Wang et al., 2021; Yan et al., 2021; Kantharaju et al., 2022). For instance, Sado et al. (2019) designed a lower-body exoskeleton to help with repetitive load-lifting and manual-handling jobs (Sado et al., 2019). A semi-active exoskeleton developed by Wang et al. (2021) was shown to reduce lower limb muscle fatigue when squatting (Wang et al., 2021). Yan et al. (2021) designed a lightweight and passive lower-limb exoskeleton that serves as a chair for workers and allows them to squat for prolonged durations (Yan et al., 2021).

In clinical settings, squatting exercises are frequently utilized in strength training and rehabilitation to help patients recover from lower extremity injuries (McGinty et al., 2000; Yu et al., 2019). The squat strengthens lower-body muscles after joint-related injuries, patellofemoral dysfunctions, ligament lesions, or ankle instability (Wallace et al., 2002; Schoenfeld, 2010; Pangan and Leineweber, 2021). Squatting is also a symmetric bilateral exercise, which can be used to strengthen muscles on one or both sides of the body, depending on the needs of the patients (Luo et al., 2021). Since individuals who perform squatting exercises for rehabilitation in clinical settings have reduced physical strength, the use of assistive exoskeletons that reduce physical effort is highly motivated. Therefore, an assistive exoskeleton can be a very beneficial device in both industrial as well as rehabilitation settings.

The ankle joint’s strength and mobility are very important for executing the squatting movement properly (Schoenfeld, 2010). During a squat performance, the ankle complex contributes significant support and aids in power generation (Hung and Gross, 1999). The importance of the ankle during squatting is supported by theoretical (McLaughlin et al., 1978; Dahlkvist et al., 1982; Robertson et al., 2008) as well as computational studies (Panero et al., 2017; Lu et al., 2020). Hence, a wearable ankle exoskeleton must effectively assist the squatting movement. An ankle exoskeleton is a lower limb wearable device that provides an assistive force to help people perform physical movements, such as walking and squatting more efficiently (Figure 2A). The device off-loads the force generated by the calf muscles, therefore lowering the metabolic energy consumed in muscle contractions (Wiggin et al., 2011; Collins et al., 2015; Jackson and Collins, 2015; Jeong et al., 2023).

### 1.2 Need for human-in-the-loop (HIL) optimization

However, the major challenge involved in exoskeleton-assisted squatting is inter-subject variability. The biomechanical movements, patterns of muscle activation, and range of motion are highly subject-specific and therefore vary across individuals (Swinton et al., 2012). To address this challenge, a personalized assistance technique was developed using the human-in-the-loop (HIL) optimization scheme (Felt et al., 2015; Zhang et al., 2017; Ding et al., 2018; Kim et al., 2019). The HIL optimization method identifies a personalized optimum parameter of the exoskeleton, which minimizes the user’s energy expenditure or metabolic cost, thus accounting for the performance variability between subjects. The metabolic cost refers to the energy expended by the human body to perform a given task (Givoni and Goldman, 1971). Metabolic energy expenditure is measured using indirect calorimetry, where either oxygen consumption or carbon dioxide production is measured and converted into units of energy (Garby and Astrup, 1987; Cunningham, 1990; Levine, 2005). Measuring the metabolic energy expenditure of an individual during a physical activity helps us investigate how the central nervous system optimizes its motor strategies to conserve energy and achieve a more efficient movement (Sousa et al., 2012). In experimental settings, it is measured using gas exchange analyzers worn by the subjects. The personalized assistance through HIL optimization method was shown to significantly reduce the metabolic cost of walking (Zhang et al., 2017; Ding et al., 2018; Kim et al., 2018; Li et al., 2020; Wen et al., 2020; Song and Collins, 2021; Gordon et al., 2022), running (Witte et al., 2020; Miller et al., 2022), and squatting (Kantharaju et al., 2022).

In a similar way, measuring the metabolic cost of exoskeleton-assisted squatting helps us identify the optimal device parameters that minimize energy expenditure and improve the efficiency of the squatting movement. In this case, the parameters are the stiffnesses, K_descending_ and K_ascending_, which represent the assistive torque pattern provided by the device. Among different torque patterns (which can also be viewed as different possible “shapes of the triangle” in Figure 2B), the best torque pattern needs to be identified such the user’s energy is minimized, which is termed “optimal assistance”. By measuring the metabolic cost using a gas exchange analyzer, the torque pattern from an exoskeleton can be optimized for each user. Kantharaju et al. (2022) showed that such a personalized assistance method is a promising approach to reduce the energy expenditure during squatting, as evidenced by a metabolic cost reduction of nearly 20% for the optimal assistance condition compared to a generic condition (Kantharaju et al., 2022).

### 1.3 Limitations of the standard HIL optimization approach

The HIL optimization method aims to minimize the metabolic cost as a cost function. Metabolic cost derived from calorimetry has been the benchmark metric for evaluating the effectiveness of exoskeleton assistance (Felt et al., 2015; Zhang et al., 2017; Ding et al., 2018; Kim et al., 2019; Kantharaju et al., 2022), and it has been used as a cost function in HIL optimization schemes. There are several limitations in using the metabolic cost, measured through indirect calorimetry (gas exchange analysis), as a cost function. The measurement of energy expenditure takes time due to slow mitochondrial dynamics (Selinger and Donelan, 2014). It typically takes at least 4–5 min to obtain an estimate for each assistance condition of the exoskeleton. The signal-to-noise ratio is low due to the presence of noise in the respiratory measurements. Furthermore, measurement through gas exchange analysis requires subjects to wear an uncomfortable mask while performing the movements. In our HIL experiments in the past, subjects have often expressed their discomfort while wearing the mask. In addition, the mask makes it hard to perform optimization outside of the lab. These disadvantages limit the practical application of calorimetry-based estimation of metabolic cost in the real world.

### 1.4 Motivation for a new HIL optimization approach

Therefore, it is important to find an alternate metric that can predict the benchmark metric, metabolic cost, in a time-efficient way and that can be measured using a comfortable wearable sensor. Jacobson et al. (2022) showed that gait symmetry derived from foot contact forces could be used to estimate the metabolic cost of walking as a rapid and comfortable measure (Jacobson et al., 2022). Using a cost function based on the novel foot pressure symmetry index within the HIL optimization scheme, eight subjects significantly reduced their energy expenditure during walking by 15% compared to the standard condition. Table 1 summarizes past research on exoskeletons designed to assist squatting movements, showing measures of metabolic cost, fatigue and balance, the type of sensors used, use of machine learning methods and the activities studied. From Table 1, the work of Jacobson et al. (2022) is the only one that shows how the alternate metric (gait symmetry) is correlated with the benchmark metric, the metabolic cost.

**TABLE 1:** Past research on exoskeletons designed to assist squatting movements. A comparison of methods used to measure metabolic cost, fatigue and balance, the type of sensors used, use of machine learning methods and the activities studied.

Reference	Physical effort (metabolic cost)	Fatigue	Balance	Sensor (s)	Machine learning method	Activity
Mohri et al. (2017)	N/A	EMG (rms)	N/A	Two EMG sensors	N/A	squat lifting
Ranaweera et al. (2018)	N/A	EMG (rms)	N/A	Two EMG sensors (Delsys, United States)	N/A	squatting, squat lifting
Sado et al. (2019)	N/A	EMG (rms)	N/A	Two EMG sensors (Shimmer Sensing Technology, Ireland)	N/A	squat lifting, carrying while walking
Wang et al. (2021)	Indirect calorimetry	EMG (rms)	N/A	Eight EMG sensors (Biometrics Limited, United Kingdom), Wearable metabolics (Cosmed, Italy)	N/A	squatting, carrying, standing, walking
Yan et al. (2021)	N/A	EMG (rms)	N/A	Four EMG sensors (Biometrics Limited, United Kingdom)	N/A	squatting
Jeong et al. (2020)	N/A	EMG (rms)	CoP variation	Six EMG sensors (Delsys, United States), Air pressure sensor tubes on shoe sole	N/A	squat lifting
Kantharaju et al. (2022)	Indirect calorimetry	EMG (rms)	N/A	Eight EMG sensors (Delsys, United States), Wearable metabolics (Cosmed, Italy)	N/A	squatting
Jeong et al. (2023)	Indirect calorimetry	EMG (muscle synergy)	N/A	Eight EMG sensors (Delsys, United States), Wearable metabolics (Cosmed, Italy)	Linear regression	squatting
Jacobson et al. (2022)	Indirect calorimetry	N/A	Gait symmetry	Foot pressure sensor (Tekscan, United States), Wearable metabolics (Cosmed, Italy)	Linear regression	walking

**FIGURE 1:**
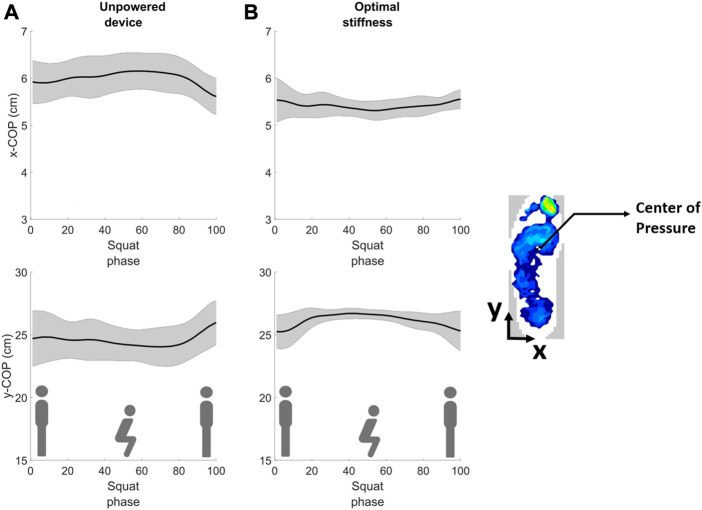
The foot Center of Pressure trajectories (in the medial-lateral direction (xCoP) and anterior-posterior direction (yCoP) for a representative subject while squatting with the device in **(A)** unpowered and **(B)** optimal assistance conditions. The CoP trajectories are shown with the variability (standard deviation) after time synchronous averaging.

In the context of physical exercises such as squatting, information about postural control and balance is important to prevent injuries from incorrect performance of the squat. Ideally, the cost function should provide such information in addition to estimating energy expenditure. Research has shown that metabolic cost can be associated with balance-related effort (Kim and Collins, 2015; Kim and Collins, 2017) suggesting that balance-related metrics can be an alternative solution. Identifying such a metric that provides information regarding posture and balance, in addition to metabolic cost, would be very valuable as it could be used for preventing injuries (or mitigating the risks of developing them) due to improper squat performance. For instance, poor postural control is associated with a higher risk of sustaining an ankle sprain (McKeon and Hertel, 2008). In this regard, the movement of the foot center of pressure (CoP) has been identified as a measure of neuromuscular control of postural balance (Han et al., 1999; Lugade and Kaufman, 2014). The center of pressure (CoP) is defined as the point on the plantar surface of the foot where the vertical ground reaction force acts (Chesnin et al., 2000). The yCoP represents the position of the center of pressure (with respect to the origin) in the anterior-posterior (or toe-heel) direction on the sole of the foot, while xCoP is the position of the center of pressure in the medial-lateral direction (shown in Figure 1). Furthermore, the CoP, as an indicator of balance, is an important evaluation metric in the clinical setting for patients and older adults (Karst et al., 2005; Ruhe et al., 2011; Mettler et al., 2015; Li et al., 2016).

In our preliminary study (Ramadurai et al., 2022), we conducted an initial analysis using previously collected data on personalized squat assistance using HIL optimization of a unilateral ankle exoskeleton (Kantharaju et al., 2022), and we found that CoP variability tended to be lower (indicating higher postural stability) for the optimal assistance condition compared to the unpowered device condition (Figure 1). Larger variability in the CoP trajectory has been associated with poorer ability to maintain balance (Abrahamova and Hlavačka, 2008). The increase in the variability of the CoP trajectories while squatting with the unpowered exoskeleton could be due to a disturbance in the natural body balance caused by wearing the exoskeleton. This may have entailed additional effort from the subject to maintain the balance of the body (Jeong et al., 2020). The reduced CoP variability in the optimal assistance condition indicates that the exoskeleton might have helped reduce the subject’s efforts to maintain postural balance, which reflects as minimized metabolic cost (Kim and Collins, 2015; Jeong et al., 2020). Based on a similar principle, Jeong et al. (2020) developed a squat assistance method for an exoskeleton by considering the minimization of CoP variation for assisting body balance during the squatting movement. (Jeong et al., 2020). However, the relationship between the CoP variation and the metabolic energy expenditure (the benchmark metric) was not investigated in their experiment.

### 1.5 Problem statement

#### 1.5.1 Research gap

To the best of our knowledge, research on development and validation of center of pressure-based metabolic cost estimation functions for HIL optimization of exoskeleton assistance has not been done.

#### 1.5.2 Objective

In this study, we aim to investigate the correlation between foot CoP features and metabolic cost and the potential for machine learning models to predict the metabolic cost of squatting using CoP features.

#### 1.5.3 Rationale for choosing machine learning

Machine learning and AI techniques are increasingly being utilized in the control of wearable exoskeletons (Baud et al., 2021; Vélez-Guerrero et al., 2021). Machine learning relies on data-driven approaches, which are robust in dealing with realistic human-exoskeleton interaction forces (Luo et al., 2021). These methods are capable of handling high dimensional data that are interrelated, as observed in human gait and exercise (Khera and Kumar, 2020). In addition, control strategies based on machine learning can address the variability between subjects (Khera and Kumar, 2020). Machine learning models also possess generalization ability, even when the size of the training dataset is small (Khera and Kumar, 2020). In experimental studies on human-in-the-loop exoskeleton optimization, the sample size is typically small (7–10 subjects), and there is inter-subject variability in physiological measurements. Hence, machine learning methods are well suited for these studies. A traditional linear regression model has the advantage of interpretability; however, it only works well if the underlying relationship between the input and output is truly linear. Machine learning methods are more suitable to capture underlying relationships that may be more complex and non-linear. Hence, we chose a machine learning approach so that the predictive model can be utilized for diverse kinds of underlying functions.

#### 1.5.4 Hypotheses

We hypothesize that balance-related features extracted from the foot CoP movement during squatting can be used to estimate the metabolic cost using machine learning. We also hypothesize that feature selection techniques can reveal important CoP features that correlate with the metabolic cost.

To test our first hypothesis, we processed, analyzed, and extracted the CoP trajectories corresponding to the squat phase for all subjects from previously collected data (Kantharaju et al., 2022). Rather than relying on a single CoP-based measure, multiple measures derived from the CoP trajectory are generally used in conjunction with each other for a more robust assessment of balance. (Baltich et al., 2014; Quijoux et al., 2021). Hence, we are interested in multiple statistical features derived from the CoP trajectories as well as the CoP velocities in both anterior-posterior (y) and medial-lateral (x) directions of the foot. The CoP velocities and statistical features were extracted from the CoP squat trajectories and fed as input to a machine learning model, where the metabolic cost was the output (to be estimated). The model was validated using unseen test data as well as the leave-one-subject-out method. To test the second hypothesis, feature selection was used to identify the features that are important for predicting the metabolic cost. Pearson’s correlation analysis was done to investigate the correlation between the important features and the metabolic cost.

## 2 Methods

### 2.1 Experimental protocol

**FIGURE 2:**
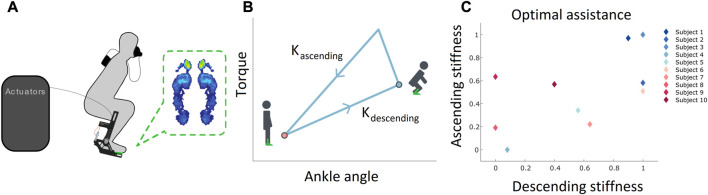
Experimental setup of exoskeleton personalization study for squatting using human-in-the-loop (HIL) optimization and its results **(A)**. Experimental setup image showing the human subject wearing the ankle exoskeleton, off-board actuators, foot pressure sensors (in green dashed box) and metabolic cost measurement device **(B)**. Ankle angle versus the desired torque trajectory for control of the exoskeleton during squatting. The K_ascending_ parameter is the proportional stiffness when the subject is performing the ascending motion from the bottom position of the squat and K_descending_ is the proportional stiffness when the subject is descending into the bottom position of the squat **(C)**. The optimal stiffness parameters were determined from HIL optimization, for each subject.

We performed a secondary data analysis of the previous experiment (Kantharaju et al., 2022) on human-in-the-loop optimization of the exoskeleton to minimize squat efforts (Figure 2). Ten healthy male subjects (age = 24.6 ± 4.0) were recruited for the experimental study. The Institutional Review Board at the University of Illinois at Chicago approved our study protocol (IRB#2020-0563). The subjects wore a tethered ankle exoskeleton on their dominant leg. Two off-board actuators (Humotech, PA, United States) were used to power the exoskeleton through a Bowden cable system. Magnetic encoders and ension load cells, which measured ankle angle and torque, respectively, were embedded within the exoskeleton. To tune the assistive torque of the exoskeleton during the ascent and descent of the squat, we used an impedance controller with two stiffness parameters, K_ascending_ and K_descending_. The assistive torque generated by the exoskeleton is proportional to the ankle angle (angle between the lower leg and the vertical axis in the sagittal plane). The stiffness parameters K_descending_ and K_ascending_ represent the slopes of the torque-ankle angle curves for the descending and ascending phases of the squat, respectively (Figure 2B).

Ankle dorsiflexion refers to the movement of flexing the foot towards the shin and knee, while plantar flexion refers to the opposite motion; i.e., the movement of the foot in a downward direction away from the knee. The squatting movement consists of the descending and the ascending phases. During the descending phase, the person moves downward into the squat position through flexion of their hips and knees and ankle dorsiflexion. During the descending phase, the ankle angle increases and reaches a maximum at the bottom position of the squat (Figure 2B). Then, during the ascending phase, the person moves upward to stand through the extension of their hips, knees, and ankle plantarflexion. During the ascending phase, the ankle angle decreases and reaches zero in the standing position (Figure 2B).

**FIGURE 3:**
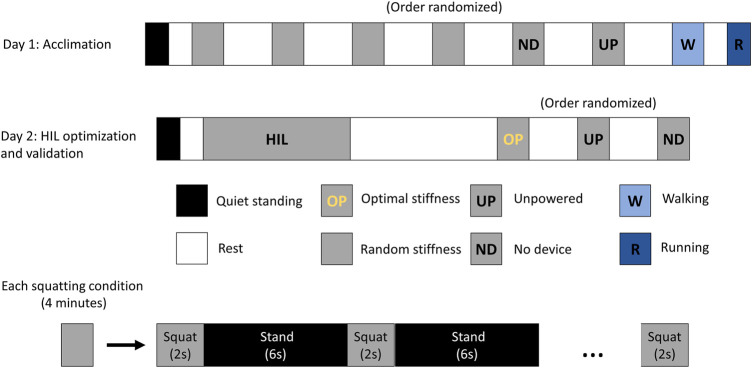
Experimental protocol for the exoskeleton personalization study for squatting. On Day 1, the subjects got acclimatized to the exoskeleton. Four different stiffness parameters were used and the corresponding metabolic cost was measured. Day 2 involved HIL optimization and validation of the optimal assistance condition. Each squatting condition lasted for 4 min in total, consisting of alternate squatting (2 s) and standing (6 s).

The experiments were conducted over 2 days. The experimental protocol is shown in Figure 3. On the 1st day, the subjects underwent an acclimation period to become familiarized with the exoskeleton emulator system. The squatting exercise was performed in four different stiffness conditions of the exoskeleton as well as the unpowered condition. For each condition of the exoskeleton, the subjects were instructed to perform squatting and standing alternately for 4 min in total. Each squat cycle lasted about 2 s, followed by 6 s of standing. The subject’s squat frequency was regulated by a metronome. The total duration of the squatting study on the 1st day was 80 min. During this time, participants performed squatting movements for a total duration of 20 min, with intervals of rest in between.

On the 2nd day, the study involved human-in-the-loop optimization of stiffness parameters for personalized assistance (Figure 2C). This was followed by a validation study in which participants performed the exercise in (i) no device, (ii) unpowered device, and (iii) optimal assistance conditions. We used data from the 1st and 2nd days for our analysis. The 2nd day’s data was used for initial analysis, where we compared the CoP variability between unpowered and optimal assistance conditions of the exoskeleton (Ramadurai et al., 2022). The 1st day’s data was used for the machine learning and foot pressure—metabolic cost correlation analysis, which are the main outcomes of this paper. The detailed description of the experimental study is provided by Kantharaju et al. (2022).

### 2.2 Metabolic cost estimation

The corresponding energy expenditure (metabolic cost) of the subject was measured using the respiratory metabolic measurement device (K5, Cosmed, Rome, Italy). The phase-plane based data-driven method was utilized to estimate the metabolic cost from the measured respiratory signals (Kantharaju and Kim, 2020).

### 2.3 Pressure measurement

The F-scan insole pressure sensor (Tekscan, MI, United States) was used to acquire foot pressure data, which consists of 3 signals: the foot pressure magnitude, the position of the center of pressure in the anterior-posterior direction (yCoP) and the position of the center of pressure in the medial-lateral direction (xCoP) of the foot. The pressure-sensing insoles were positioned above the insoles of the subjects’ shoes. Prior to data collection, a standard step calibration was performed to calibrate the pressure data for each individual. The sampling rate was set to 50 Hz. Following the calibration, data collection commenced.

### 2.4 Data analysis

For the preliminary analysis (Figure 1), six subjects’ foot pressure data from the validation study on the 2nd day of the experiment were selected based on data quality and analyzed (Ramadurai et al., 2022). The CoP variability at the optimal assistance condition was compared with the unpowered device condition. The CoP variability (standard deviation) was used as it has been found to be a reliable and consistent measure of postural equilibrium (Geurts et al., 1993; Le Clair and Riach, 1996; Palmieri et al., 2002; Quijoux et al., 2021). Lower values indicate better postural control.

#### 2.4.1 Data analysis steps

**FIGURE 4:**
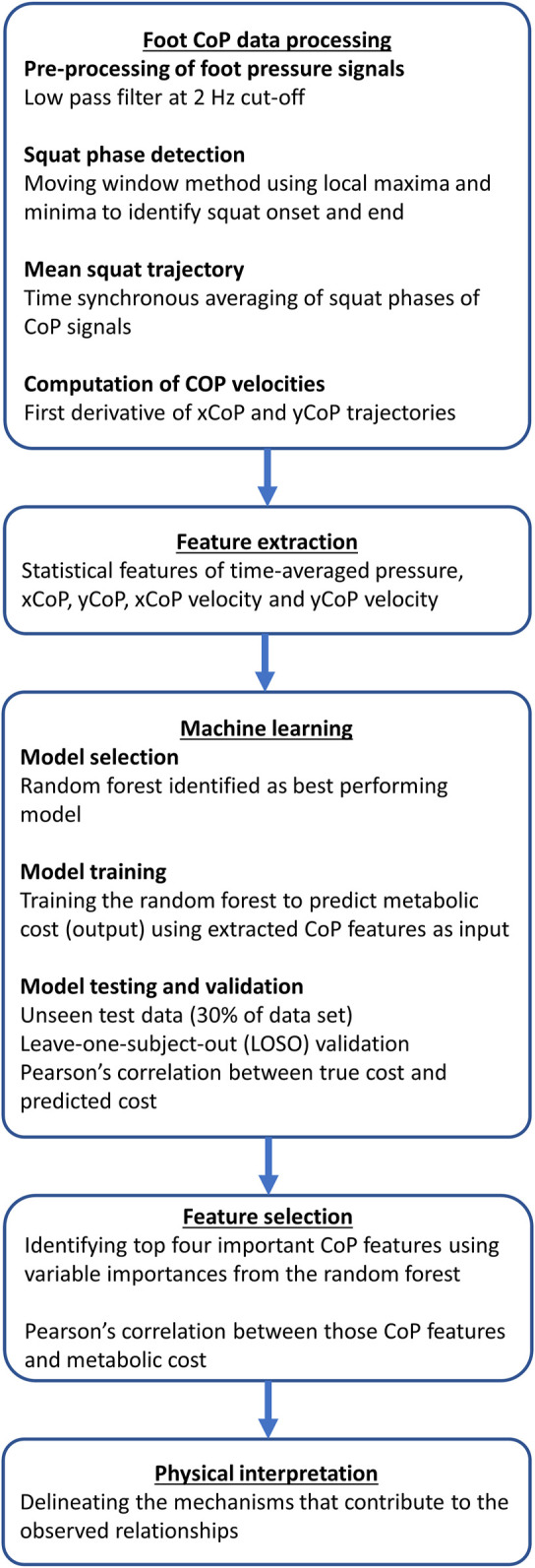
Flowchart showing the steps involved in processing and analyzing foot pressure data for exoskeleton-assisted squatting. Features extracted from the processed foot pressure signals for each assistance condition were used to train a machine learning model (random forest) to predict the corresponding metabolic cost. Feature selection was done to identify the CoP features that were most important in predicting the metabolic cost.

For the machine learning analysis, we used the data from the 1st day of the experiment. Essentially, we instructed the subjects to perform squatting movements periodically for a fixed duration of 4 min while wearing a powered exoskeleton and collected the foot CoP data (from a pressure sensing insole placed inside the shoe) and the metabolic energy consumption (using indirect calorimetry). Foot CoP and metabolic cost data were collected for different assistive torque patterns of the exoskeleton. We then extracted relevant features from the CoP data, such as the mean, standard deviation, minimum and maximum of pressure, xCoP, yCoP, xCoP velocity and yCoP velocity during the squat phase. Then, we tried to find the mapping between foot CoP features (input) and the metabolic cost (output) using a machine learning model, the random forest. We split the data set into training and test sets using a 70%–30% split. After training the model, we tested the model’s prediction performance using unseen test data (30% of data held out from the original split). The model’s prediction accuracy was determined based on the correlation between the true metabolic cost measured through calorimetry and the metabolic cost predicted by the random forest model. We also tested the model’s performance on unseen subjects using leave-one-subject-out (LOSO) validation. Using the feature importance values, we identified the most important features with predictive power to estimate the metabolic cost. Pearson’s correlation analysis was done to estimate the individual correlation coefficients between the important features and the metabolic cost. These steps are summarized in Figure 4 and explained in more detail below.

#### 2.4.2 Foot CoP data processing

The subjects performed squatting under different stiffness conditions of the exoskeleton and the corresponding metabolic cost and foot pressure data were measured. The data of five subjects who had clear foot pressure waveforms were chosen for analysis. The last 2 min of the signals were considered for the analysis, in order to control for adaptation effects during the initial squats. The pressure, yCoP, and xCoP signals were processed using a low pass filter at 2 Hz. The signals were analyzed using MATLAB. The squat onsets and squat phase were identified using the peaks and local extrema in the pressure signal using a moving window technique. The squat phases of the signals were averaged using time synchronous averaging. The CoP trajectories after time synchronous averaging are shown in Figure 1.

#### 2.4.3 Feature extraction

The xCoP and yCoP velocities were obtained by calculating the first derivative of the xCoP and yCoP trajectories, respectively. The statistical features (minima, maxima, mean, and standard deviation) of the pressure, yCoP, xCoP, yCoP velocity, and xCoP velocity signals were extracted. The extracted features were used as predictors of metabolic cost in the machine learning model.

#### 2.4.4 Model selection

A decision tree is a machine learning method that partitions the feature space into a number of non-overlapping regions with similar output values using splitting rules. In this case, the feature space is represented by the set of foot CoP features while the output values are the metabolic cost magnitudes. The random forest is an ensemble learning technique that combines the results of a large number of uncorrelated decision trees, trained on random subsets of features to make a prediction (Breiman, 2001). Minimizing the correlation between individual trees reduces the prediction error. As a data-driven model, the random forest has important advantages such as robustness to noise and overfitting. It also gives useful estimates of variable importance, which can be utilized for feature selection (Kulkarni and Sinha, 2012; Fawagreh et al., 2014). We chose the random forest for our dataset because it showed the best performance among the other models tested such as k-nearest neighbors, support vector classifier and linear discriminant analysis.

#### 2.4.5 Model training

The features extracted from the foot pressure data for each stiffness condition of the exoskeleton were fed as input to the random forest regressor. The corresponding metabolic cost was the response. The machine learning analysis was done using Python (scikit-learn, RRID: SCR_002577). The dataset was divided into training and testing sets using a 70%–30% split. The training set was used for tuning the model’s hyperparameters, such as the number of trees, maximum depth of each tree, and minimum number of samples per leaf. A grid search method was used, and the hyperparameters were selected based on five-fold cross-validation. In addition to the different stiffness conditions, each subject’s data for the no-device and unpowered device conditions were also included for training the model. This procedure was repeated for all five subjects. Adding the data from the no-device and unpowered device conditions helped the model learn the variability in measured values across different subjects.

#### 2.4.6 Model testing and validation

The testing set (30% of the data) was held out as new data unseen by the model. The accuracy of the model’s predictions was evaluated using Pearson’s correlation coefficient between the true metabolic cost (from indirect calorimetry) and the metabolic cost predicted by the random forest model. Correlation values above 0.8 are considered to be very strong. (Taylor, 1990; Evans, 1996). The statistical signficance is reported at alpha = 0.05. The model’s prediction accuracy on unseen subjects was also investigated using leave-one-subject-out validation. Since five subjects’ data were available, one subject was selected as the unseen test subject and the model was trained using the data from the remaining four subjects. In this manner, the model was tested on every subject.

#### 2.4.7 Feature selection

Feature selection was performed using variables importances returned by the random forest model. The top four important CoP features were investigated for their correlation with the metabolic cost using Pearson’s correlation analysis.

#### 2.4.8 Physical interpretation

We then interpreted our results from a biomechanical perspective, to delineate the mechanisms that could be contributing to the observed results.

## 3 Results

### 3.1 Model performance

**FIGURE 5:**
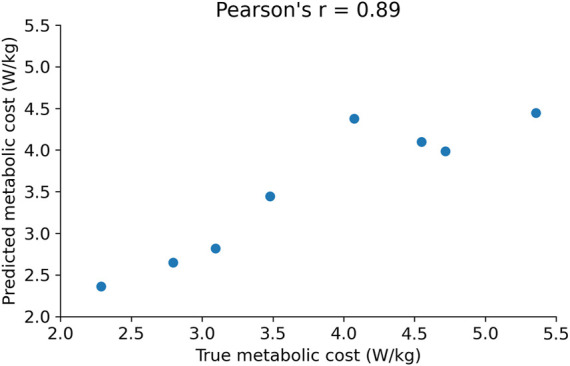
Correlation between the true metabolic cost and the cost predicted by the random forest model using foot pressure features.

After training the random forest model, we tested its accuracy in predicting the metabolic cost on unseen data from the test set. The prediction error (mean absolute error; MAE) on the test set was ± 0.55 W/kg (percentage error: 11%) (Table 2). Pearson’s correlation analysis showed a statistically significant and high correlation (r = 0.89; *p* < 0.01) between the true metabolic cost and the metabolic cost predicted by the random forest model (Figure 5), as r > 0.8 is considered to be very strong. (Taylor, 1990; Evans, 1996).

**TABLE 2:** Metabolic cost prediction performance of the random forest regressor on the hold-out test dataset and unseen test subjects, using the LOSO (leave-one-subject-out) method.

Test data	Mean absolute error in predicted metabolic cost (W/kg)	Mean absolute percentage error (%)
	Hold-out test set validation	
70%–30% split of entire dataset	0.55	11.1
	Leave-one-subject-out validation	
Subject 1	0.45	13.1
Subject 2	0.75	38.2
Subject 3	0.42	9
Subject 4	0.07	2.7
Subject 5	0.32	6.8
Average	0.40	13.9
Median	0.42	9

The results of the leave-one-subject-out validation are shown in Table 2. The average error for the unseen subject was ± 0.40 W/kg. The average percentage error for the unseen subjects was 13.9%. However, Table 2 shows that the percentage error for one subject (Subject 2) is very high and is influencing the mean. Hence, the median percentage error for the subjects was also calculated and found to be 9%.

### 3.2 Feature selection and correlation analysis

**FIGURE 6:**
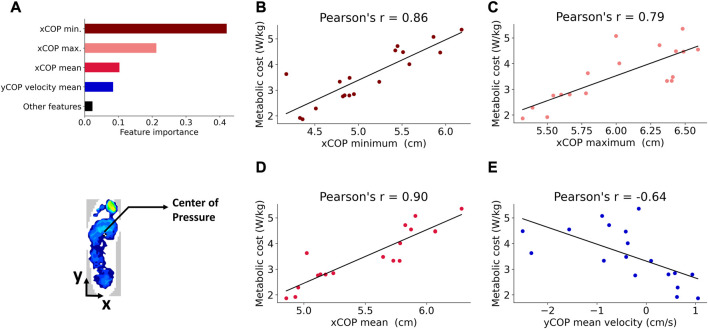
**(A)** Top four important features returned by the random forest: minimum, maximum and mean of the xCoP squat trajectory, and yCoP mean velocity. The importance of the remaining features was below the value indicated by the black bar **(B)**. Correlation between the xCoP minimum and the metabolic cost **(C)**. Correlation between the xCoP maximum and metabolic cost **(D)**. Correlation between the xCoP mean and metabolic cost **(E)**. Correlation between the yCoP mean velocity and metabolic cost.

Feature selection was based on the feature importance returned by the random forest algorithm. The top four most important features were the minimum, maximum and mean values of the xCoP squat trajectory and the mean velocity of the yCoP trajectory, as shown in Figure 6A. The important features determined by the random forest model were investigated further through correlation analysis, and the correlations were found to be statistically significant. The Pearson’s correlation plots for the top four important features, namely, xCoP minimum (r = 0.86, *p* < 0.001), xCoP maximum (r = 0.79, *p* < 0.001), xCoP mean (r = 0.9, *p* < 0.001) and yCoP mean velocity (r = −0.64, *p* < 0.01), are shown in Figures 6B–E. The mean, minimum and maximum values of the xCoP trajectory showed an increasing trend with increasing metabolic cost. The yCoP mean velocity showed a decreasing trend with metabolic cost.

Due to the strong linear correlations observed for the important features, we have also expressed the relationship between the highly correlated CoP features and metabolic cost in terms of the traditional method, the multiple regression equation, which is simple and interpretable.

Metabolic cost=−7.454+1.369 xCoP mean+0.522 xCoP minimum+0.129 xCoP maximum

(Units: Metabolic cost is in W/kg; xCoP mean ; xCoP minimum ; xCoP maximum are in cm)

## 4 Discussion

In this paper, we analyzed the foot pressure data obtained from the study (Kantharaju et al., 2022) on human-in-the-loop optimization of an ankle exoskeleton to minimize the physical effort (metabolic cost) of squatting using a machine learning method, random forest regression analysis. Based on our preliminary results (Ramadurai et al., 2022), we hypothesized that metabolic cost can be estimated from CoP-derived metrics using machine learning methods like the random forest for improved time-efficiency and user comfort in HIL optimization. We also hypothesized that the random forest can reveal important CoP features that are correlated with the metabolic cost of squatting. We found that foot pressure features obtained from insole-based pressure mapping systems can be used to predict the metabolic cost using machine learning techniques. The random forest regressor was able to predict the metabolic cost with an error of 0.55 W/kg on unseen foot pressure data. The model was also validated using LOSO (leave-one-subject-out) and the average error was 0.4 W/kg (percentage error: 9%) on the unseen subject. The statistically significant high correlation (r = 0.89) between the true metabolic cost and predicted cost indicates that the random forest model was able to capture the underlying relationship between the metabolic cost and CoP features, supporting the first hypothesis. The important features of the input data, such as the mean, minimum and maximum of xCoP squat trajectory and the yCoP mean velocity, were obtained through feature selection from the random forest model. These features were also significantly highly correlated with the metabolic cost, supporting the second hypothesis.

**FIGURE 7:**
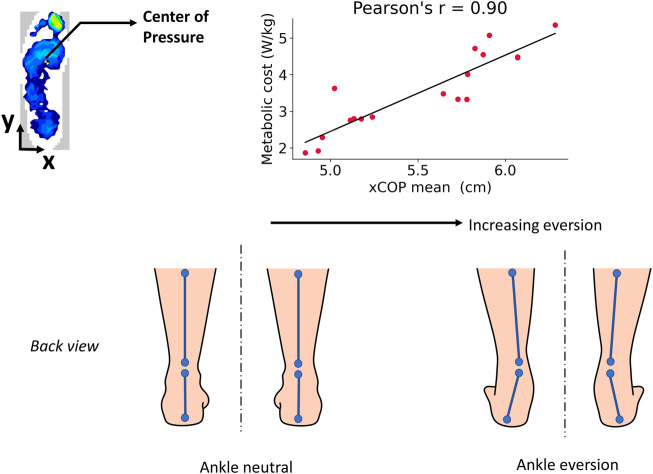
Interpretation of the main results. Increasing ankle eversion (outward ankle roll) was associated with higher metabolic costs, indicating a sub-optimal squatting strategy at those assistive torques of the exoskeleton.

A high positive correlation was found between the metabolic cost and the statistical features of the xCoP squat trajectory: xCoP mean (r = 0.9), xCoP minimum (r = 0.86), xCoP maximum (r = 0.79). In other words, the metabolic cost could be minimized when the mean and extrema of the xCoP squat trajectory were lower. The xCoP represents the position of the center of pressure in the medial-lateral direction of the foot. From Figures 6B–D, a larger value of xCoP indicates that the CoP moved further in the medial direction of the foot while the subject was squatting. The interpretation of this result is explained as follows. All subjects were instructed to squat the same way. In the standing position, they stood with their feet apart (shoulder width apart). While squatting, their knees had to be aligned with their toes during both the descent and ascent. Based on our data analysis, we found for certain assistive torque patterns of the exoskeleton, the subjects did not maintain their knee-toe alignment while squatting. Their knees collapsed inwards, along with an outward roll of the ankle, resulting in the xCoP shifting towards the medial direction. The metabolic cost was found to be higher with increasing medial shift of the xCoP. In biomechanics parlance, the inward collapsing of the knee is termed valgus movement while the outward roll of the ankle is called eversion. A higher ankle eversion, reflected as increased xCoP medial shift, is known to be the cause of chronic lower limb injuries (Lee et al., 2010; Whitting et al., 2016; Pangan and Leineweber, 2021). Hence, the metabolic energy expenditure might have increased with greater outward roll of the ankle because it is a non-optimal squatting strategy associated with the risk of injury. We posit that at suboptimal assistive torques, the knees “wobbled” out of alignment with the toes and the ankle rolled outwards, causing the subjects to exert more effort to maintain their squat posture and balance, which manifested as increased metabolic cost. At the optimal assistive torque, the subjects were able to execute the squats smoothly, with reduced ankle roll and better knee-toe alignment, expending less energy (Figure 7).

The medial shift of the CoP indicates greater ankle eversion and knee abduction, which is indicative of a suboptimal squatting strategy (Chiu et al., 2013; Snarr and McGinn, 2015; Pangan and Leineweber, 2021) and resulted in increased metabolic cost (Houdijk et al., 2015). It is well-known that a high degree of ankle mobility and strength is necessary to facilitate postural balance and control during the descending and ascending motions of the squat (Schoenfeld, 2010). Insufficient stability and strength in the ankle joints could lead to the hips, knees, and feet negatively compensating, leading to muscular imbalance or injury (Cook, 2003; Kendall et al., 2005; Snarr and McGinn, 2015). The squat movements are facilitated by ankle dorsiflexion and plantarflexion, which maintain postural stability and limit inversion and eversion at the foot (Schoenfeld, 2010). A higher ankle eversion (indicated by the movement of the CoP towards the medial direction) has been associated with the etiology of chronic lower limb injuries (Lee et al., 2010; Whitting et al., 2016; Pangan and Leineweber, 2021). Eversion causes the ankle to collapse inward during the squat. This is typically accompanied by an inward collapsing of the knee joints and hip adduction, known as a valgus movement, which increases the risk of injury due to muscular imbalances of the inner and outer thigh (Snarr and McGinn, 2015). Valgus movement, with eversion at the foot, is associated with reduced strength of the hip abductors (Snarr and McGinn, 2015; Rinaldi et al., 2022). Claiborne et al. (2006) found that individuals with greater strength in the knee extensors, knee flexors and hip abductors were less likely to collapse inwards at the knee joints while squatting (Claiborne et al., 2006). Excessive hip adduction during knee valgus is known to be a common risk factor for several acute lower extremity injuries, including the ACL (anterior cruciate ligament) injury (Griffin et al., 2000; Rinaldi et al., 2022). Therefore, it is possible that the metabolic cost of squatting increases with greater eversion of the foot because it is a non-optimal squatting strategy that increases the risk of injury.

From the analysis results, we infer that the xCoP trajectory during the squat can be utilized not only to estimate the metabolic cost but also to detect sub-optimal squat performance that could help prevent lower limb injuries. The features of the xCoP trajectory can be easily obtained from a pressure-sensing insole worn inside the shoe. It is a comfortable and portable wearable sensor and allows for both rapid estimation of metabolic cost and injury risk due to ankle eversion. Hence, the possibility of using xCoP features as an alternative cost function in human-in-the-loop exoskeleton optimization seems promising. Furthermore, considering postural balance metrics when designing a control method for exoskeletons is important because the natural body balance could be perturbed by the wearing of the exoskeleton and require additional effort from the user to regain their balance (Jeong et al., 2020; Luo et al., 2021).

The correlation between the metabolic cost and yCoP mean velocity was found to be moderate and negative (r = −0.64). The yCoP velocity is a measure of the displacement of the CoP in the anterior-posterior direction of the foot. Positive values indicate movement of the CoP toward the toe (anterior direction), while negative values indicate CoP movement toward the heel (posterior direction). The absolute value reflects the magnitude of the displacement. In this study it was found that displacements of the CoP in the anterior direction during squatting, i.e., towards the toe, were correlated with lower metabolic costs, while CoP displacements towards the heel were associated with higher metabolic costs. Ishida et al. (2022b) found that the yCoP position is a significant predictor of the knee extensor moment contribution during a squat (Ishida et al., 2022b). Squatting with the CoP position shifted in the anterior direction resulted in a significantly lower knee extensor moment compared to a posterior CoP shift. (Ishida et al., 2022a). Perhaps this could explain why lower metabolic costs were correlated with CoP displacements in the anterior direction of the foot.

Our approach shows how machine learning and feature selection applied to balance-related measures based on foot center of pressure can offer insights into how subtle changes in movement patterns can affect performance. Previous works on human-in-the-loop optimization assessed performance by measuring metabolic cost using respiratory measures (indirect calorimetry) (Felt et al., 2015; Zhang et al., 2017; Ding et al., 2018; Kim et al., 2019; Kantharaju et al., 2022). From such past works, it is known that certain assistive torque patterns of the exoskeleton are energetically more economical, but the pathways through which the nervous system adapts to the exoskeleton to effect such an efficient movement is unknown. Our research shows how CoP can act as an external “signature” of how the nervous system internally coordinates movement patterns that result in smooth and efficient movements that are metabolically economical. Data-driven methods such as machine learning, together with feature engineering and feature selection, can identify these signatures of efficient movement from CoP data and predict the optimal condition before it manifests in the respiratory signals. Hence, such a predictive model can estimate the metabolic cost in a shorter span of time, leading to a much faster optimization process, together with the advantage of user comfort.

### 4.1 Managerial implications

In both industrial as well as clinical settings, the CoP-based assistance optimization can potentially be used to reduce energy expenditure and fatigue during repetitive squatting, with practical advantages over the current HIL optimization method. (i) *Optimization time*: The results of our study show that foot CoP features are useful in rapidly estimating the metabolic cost of squatting for human-in-the-loop optimization of exoskeleton assistance. Using the indirect calorimetry method, it takes at least 4 min to obtain a reasonable estimate of metabolic cost, whereas using the CoP-based prediction method, the metabolic cost can be estimated within 2 min. The optimization process using the standard approach takes about 20 min in total, while it can be done in 10 min using our proposed approach. Thus, the CoP-based estimated method could potentially reduce optimization time by about 50%. (ii) *User comfort*: Furthermore, the CoP data can be obtained from a non-invasive, comfortable pressure sensor worn inside the shoe; hence, it can be used outside of the lab, unlike the respiratory measure. This fast cost estimation with a comfortable foot pressure sensor may enable personalization outside of the lab. *(iii) Injury risk mitigation*: In addition, being an index of postural control and balance, the CoP can indicate improper squat performance and help reduce the risk of injury. For example, if excessive medial shift of the xCoP is observed, it indicates ankle roll (eversion) during squatting and the individual can be guided to correct their squat technique.

### 4.2 Limitations

The study was conducted with young and healthy male participants. There might be differences in the CoP trajectories of older adults and patients who have reduced physical strength. In that case, we can conduct another data analysis as there might be additional features of the CoP trajectories that are important. Another limitation is that due to the signal noise in the sensor, we were able to use only five subjects’ data. However, the relatively high Pearson’s correlation coefficient indicates that this study’s outcomes can be useful for the optimization process.

### 4.3 Conclusion

In conclusion, the features of the CoP trajectory were found to be useful in predicting the metabolic cost of exoskeleton-powered squatting using machine learning methods, as indicated by a high correlation between the true cost and predicted cost. In particular, the xCoP trajectory features related to ankle inversion and eversion were found to have a high positive correlation with metabolic cost. Increased ankle eversion (outward ankle roll) was associated with higher metabolic cost. Higher ankle eversion has been linked with an increased risk of developing chronic lower limb injuries, Hence, the xCoP trajectory features can be used to estimate both the metabolic cost of squatting as well as the risk of developing injuries due to improper squat performance. Our proposed method of using a CoP-based cost function in human-in-the-loop optimization offers multiple advantages, such as reduced estimation time, injury risk mitigation, and enhanced user comfort, and importantly makes it possible to use human-in-the-loop optimization outside of the lab.

For our future work, we plan to conduct human subject experiments to test our CoP-based metabolic cost prediction model in real time human-in-the-loop optimization of the ankle exoskeleton assistance to minimize the user’s physical exertion during squatting.

## Data Availability

The de-identified data supporting the conclusion of this article will be made available by the authors, without undue reservation.
